# Supplementary material for: Disrupted Mitochondrial and Metabolic Plasticity Underlie Comorbidity between Age-Related and Degenerative Disorders as Parkinson Disease and Type 2 Diabetes Mellitus
Source: Antioxidants (Basel). 2020 Oct 30;9(11):1063. doi: 10.3390/antiox9111063 (PMC7693963; doi:10.3390/antiox9111063)
Supplement: Supplementary file 1 [file antioxidants-09-01063-s001.pdf]

antioxidants

Article

Disrupted mitochondrial and metabolic plasticity underlie comorbidity between age-related and degenerative disorders as Parkinson disease and Type 2 Diabetes Mellitus

Juárez-Flores DL[1,2,*], Ezquerra M[3,4], Gonzàlez-Casacuberta[1,2], Ormazabal A[2,5], Morén C[1,2], Tolosa E[3,4,6], Fucho R[7,8], Guitart-Mampel M[1,2], Casado M[2,5], Valldeoriola F[3,4,6], de la Torre-Lara J[1,2], Muñoz E[3,4,6], Tobías E[1,2], Compta Y[3,4,6], Garcia-Garcia FJ[1,2], Garcia-Ruiz C[7,8,9], Fernandez-Checa JC[7,8,9], Marti MJ[3,4,6], Grau JM[1,2], Cardellach F[1,2], Artuch R[2,5], Fernández-Santiago R[3,4], Garrabou G[1,2,*].

[1] Laboratory of Muscle Research and Mitochondrial Function, Institut d'Investigacions Biomèdiques August Pi i Sunyer (IDIBAPS), Faculty of Medicine and Health Science-University of Barcelona (UB), Department of Internal Medicine-Hospital Clínic of Barcelona (HCB), 08036 Barcelona, Spain
[2] Centro de Investigación Biomédica en Red (CIBER) de Enfermedades Raras (CIBERER), 28029 Madrid, Spain.
[3] Laboratory of Parkinson Disease and Other Neurodegenerative Movement Disorders, IDIBAPS, Institut de Neurociències, UB, 08036 Barcelona, Catalonia.
[4] Centre for Networked Biomedical Research on Neurodegenerative Diseases (CIBERNED CB06/05/0018), 28029 Madrid, Spain.
[5] Department of Clinical Biochemistry, Institut de Recerca de Sant Joan de Deu, Esplugues de Llobregat, Barcelona, Spain.
[6] Parkinson disease and Movement Disorders Unit, Neurology Service, HCB, IDIBAPS, UB.
[7] Cell Death and Proliferation, Instituto de Investigaciones Biomédicas de Barcelona (IIBB), Consejo Superior Investigaciones Científicas (CSIC), Barcelona, Spain.
[8] Liver Unit-HCB, IDIBAPS, 08036 Barcelona and CIBER de Enfermedades Hepáticas y Digestivas (CIBEREHD), 28029 Madrid, Spain.
[9] USC Research Centre for ALPD, Keck School of Medicine, Los Angeles, CA 90033, USA.
* Correspondence: garrabou@clinic.cat (GG), +34 2275400 ext 2907; dian.juarez@icloud.com (D.L.J.-F).

Received: date; Accepted: date; Published: date

<u>*Mitochondrial oxygen consumption (Seahorse)*</u>

Oxygen consumption rates (OCRs) were measured with Agilent Seahorse XF24 Analyzer Mitostress Test (Seahorse Bioscience, www.seahorsebio.com), according to manufacturer's protocol. Briefly, 30,000-35,000 fibroblasts/well were seeded by quadruplicate in customized 24-well Seahorse cell culture plates and kept overnight in 250 µl of either 25mM or 5mM glucose medium. Growth medium was then removed, and wells were washed once with Seahorse XF Base Medium (Seahorse Bioscience) containing 10 mM Glucose, 1 mM Sodium Pyruvate and 1 mM Glutamine. Plates were incubated in this media for 30 min at 37 °C without CO_2. The bioenergetic profile was measured obtaining the OCRs under basal conditions and after the addition of oligomycin, carbonyl cyanide-4-(trifluoromethoxy) phenylhydrazone (FCCP) and rotenone-antimicyn (all reagents from Sigma-Aldrich). OCR values were normalized to total cell protein content and reported as pmol/min*ug protein.

Mitoworking capacity was measured as the ratio between the Basal Respiration and the Maximal respiration.

Despite using identical cell number for all analyses, results were additionally normalized by total protein and mitochondrial content through the CS activity and were expressed as pmol O_2/min*µg protein*CS activity.

<u>*Mitochondrial oxygen consumption (Oroboros)*</u>

Mitochondrial respiration was measured in parallel by high-resolution respirometry using Oroboros™ Oxygraph-2K® (Innsbruck, Austria) in permeabilized fibroblasts, following manufacturer protocols (Pesta and Gnaiger 2012). Briefly, 1 million of living fibroblasts were obtained and resuspended in ice-cold respiration MiR05 medium (0.5 mM EGTA, 3 mM $MgCl_2$, 60 mM K-lactobionate, 20 mM taurine, 10 mM KH_2PO_4, 20 mM HEPES, 110 mM sucrose and 0.1% BSA (w/v), pH 7.1). Data recording and analysis were performed using DatLab software v5.1.1.9 (Oroboros Instruments), following manufacturer's protocol. The oxygen consumption was measured using the same procedure as in the Seahorse Mitostress test: Firt measuring the "basal" respiration, followed by complete blockage of Complex V (1uM Oligomycin), titration with sequential doses of 0.5uM FCCP until a maximal respiration was achieved and finally blocking of Complex I and III (to measure non-mitochondrial oxygen consumption) through the addition of 0.05uM rotetone + 0.05uM antimycine.

Despite using identical cell number for all analyses, results were additionally normalized by total protein and mitochondrial content through the CS activity and were expressed as pmol O_2/min*µg protein*CS activity.

© 2020 by the authors. Submitted for possible open access publication under the terms and conditions of the Creative Commons Attribution (CC BY) license (http://creativecommons.org/licenses/by/4.0/).
